# Occupational exposure to blood borne pathogens among healthcare workers: a cross-sectional study of a registry in Colombia

**DOI:** 10.1186/s12995-015-0088-z

**Published:** 2015-12-16

**Authors:** Carlos Pérez-Diaz, Omar-Javier Calixto, Álvaro A. Faccini-Martínez, Juan S. Bravo-Ojeda, Carlos A. Botero-García, Erika Uribe-Pardo, Yesid F. Mantilla-Florez, Fabian Benitez, Ada Duran, Johana Osorio

**Affiliations:** Infectious Diseases Department, Clinica Marly, Bogotá, Colombia; Infectious Diseases Department, Hospital de la Samaritana, Bogotá, Colombia; Servicios y asesorías en infectología (SAI), Calle 50 # 13-62, 110231420, Bogotá, Colombia; School of Medicine, Universidad de la Sabana, Chia, Colombia; School of Medicine, Universidad Militar Nueva Granada, Bogotá, Colombia; School of Medicine, Fundación Universitaria Navarra, Neiva, Colombia

**Keywords:** Health care worker, Blood borne pathogen, Occupational exposure

## Abstract

**Background:**

Occupational exposure to blood borne pathogens caused by percutaneous injuries or mucosal contamination is frequent among Healthcare Workers (HCW).

**Methods:**

A cross-sectional analysis of HCW with an occupational exposure to blood reported to professional risk insurance agencies between 2009 and 2014 was performed. Comparisons between groups according to exposure level (mild, moderate, and severe) were evaluated.

**Results:**

Two thousand, four hundred three reports were classified according exposure as mild 2.7 %, moderate 74.8 %, severe 21.9 %. Factors related: health sciences student with mild exposure events [adjusted odds ratio (AOR) 11.91, 95 % CI 5.13–27.61, *p* < 0.00001], and physician with moderate exposure events (AOR 1.90, 95 % CI 1.17–3.07, *p* = 0.009). Factors inversely related: physician with severe exposure events (AOR 0.54, 95 % CI 0.32–0.91, *p* = 0.02) and health sciences student with moderate exposure events (AOR 0.08, 95 % CI 0.04–0.15, *p* < 0.00001). It was found an important relationship between severe events with infectious diseases specialist assessment, and follow-up adherence. Additionally, a case of Human Immunodeficiency Virus seroconversion was presented (0.0004 %), no other seroconversions were observed.

**Conclusions:**

Occupational exposure events must be managed according to established protocols, but adherence failure was evident with the exception of severe exposure cases. Thus, interventions to enhance occupational safety are required. Occupation must be considered as a risk factor during initial assessment of events.

## Background

Health Care Workers (HCW) refers to any person that performs a paid or non-paid labor activity in health care [1, 2]. This population is potentially exposed to infectious materials as corporal fluids and contaminated medical devices and surfaces. HCW include physicians, paramedics, nurses, dentists, technicians, students, and assistants whose role is related directly with health care. Additionally the personnel that is indirectly related with patients attention can also be exposed to blood or other fluids, as it is the case of cleaning, security, maintenance and volunteer personnel [1, 2].

Biohazard event is defined as the exposure to blood, tissues or any other potentially infectious fluid [1], one of the most obvious occupational risks in HCW daily practice and produces anxiety among them [3]. Exposure to blood borne pathogens caused by percutaneous injuries or mucous membranes contamination is a frequent event. It is considered, seroconversion risk for blood borne viruses are; 1–6 % to 22–31 % for Hepatitis B Virus (HBV) depending on serological status of the source, 1.8 % for Hepatitis C Virus (HCV), and 0.3 % for the Human Immunodeficiency Virus (HIV) [4]. Worldwide, the cases of infection associated to occupational activity in HCW represents 39 % of the cases of infection by HCV, 37 % of the cases of infection by HBV, and 4.4 % of the cases of infection by HIV [5]. For the region of the Americas it is considered that 57,000, 61,000 and 23,000 events occur with a positive source to HCV, HBV and HIV, respectively [5].

Universal precaution recommendations have been created for prevention, especially needles and sharp objects injuries, as well as preventing contact of fluids with mucous membranes [6].

In Colombia once the exposure event occurs the HCW must contact their insurance agency in order to be advice of initial treatment and risk assessment in the emergency room or presenting to a specialized consultation service. But there is not an integrated national record of these events, for that reason the objective of this study is to analyze the reports of exposure to blood and corporal fluids of five professional risk insurance companies in Colombia between years 2009 and 2014, as well as determining the factors associated to mild, moderate, and severe exposure classification.

## Methods

### Study design

The study was an epidemiological, retrospective, record-based review. This study included registries of Colombian HCWs who presented an episode of exposure to blood borne pathogens caused by percutaneous injuries or mucous membranes contamination reported to five professional risks insurance companies and evaluated in Servicios y Asesorías en Infectología (SAI), which performed the occupational exposure initial management and follow-up, between years 2009 and 2014. The present study protocol was approved by the Institutional Review Board, SAI.

The sample size was calculated taking into account the formula for this type of study based on expected prevalence of occupational exposure events in a local teaching hospital from Colombia (31.6 %) [31]. Based on an alpha risk of 5 % with a confidence level of 99 % and a two-sided configuration. As a result, the estimated sample size was 2294 subjects.

Needle puncture or sharp object injuries and cutaneous or mucosal splashes were surveyed by reports of exposures assessed in a specialized infectious diseases agency. The follow up consisted in guaranteeing and guiding the medical advice, and treatment of cases according to their classification in mild, moderate or severe exposure events. Sociodemographic, clinical and laboratory data were obtained from the event registries and double-check with medical registries by two investigators in order to control bias. These data was registered in an electronic database.

### Statistical analysis

In the first place, a univariate descriptive analysis was performed. Categorical variables were analyzed through frequencies. The Kolmogorov-Smirnov test was performed to evaluate the normality. Parametric data is expressed with the mean and standard deviation (SD), and non-parametric data is described with median and interquartile range (IQR).

Subsequently, a bivariate analysis was performed to establish association between demographic and clinical characteristics of the patients. Parametric values were analyzed by chi-square test or Fisher exact test. A value of *p* ≤ 0.05 was considered as significant.

A multiple logistic regression was considered by taking the exposure level classification as independent variables. As dependent factors: statistically significant associations in the bivariate analysis were used, as additional variables described in the literature.

The adequacy of the logistic models was evaluated through Hosmer-Lemeshow goodness-of-fit test. The Nagelkerke R2 was used to estimate the percentage of variance explained by the model. The adjusted odds ratio (AOR) was calculated with confidence intervals of 95 % (CI). Wald statistic test was used to evaluate the significance of different logistic regression coefficients for each independent variable. A step by step progressive elimination approach was used. Statistical analyses were made in the statistical program IBM SPSS Statistics 22.

## Results

Characteristics of the 2403 participants, 82.4 % were females, with a mean age between 29.5 ± 9.2 years. Regarding their origin, 69.8 % came from urban areas, the remaining percentage of cases occurred in rural areas. Biohazard events were classified as mild in 2.7 %, moderate in 74.8 % and severe in 21.9 %. The main mechanisms were: needle puncture (hollow needle, blunt needle, or unspecified needle) 86.5 %, mucocutaneous splash 7.9 %, and injuries with contaminated sharp object 5.6 %. For further description of demographical data and injury mechanisms, see Table 1.

**Table 1:** Characteristics of the health care workers with occupational exposure

Sociodemographic characteristics	Mean ± SD	Median, IQR
Age (y)	29.5 ± 9.2	27, 28.8–48.3
	%(n/N)	
Female	82.4 (1980/2403)	
Urban area	69.8 (1677/2402)	
Occupation	%(n/N)	
Nursing	47.5 (1141/2403)	
Physician	13.9 (334/2403)	
Student	13.1 (315/2403)	
Cleaning personnel	9.6 (230/2403)	
Odontology	7.3 (175/2403)	
Bacteriology	4.9 (118/2403)	
Surgical instrumentation	2.6 (62/2403)	
Respiratory therapy	1.1 (27/2403)	
Contact Mechanism	%(n/N)	
Hollow needle	53.0 (1272/2403)	
Unspecified needle	22.8 (548/2403)	
Blunt needle	10.7 (256/2403)	
Mucocutaneous splash	7.9 (190/2403)	
Lancet puncture	4.1 (99/2403)	
Scalpel injury	1.3 (31/2403)	
Contaminated glass injury	0.2 (5/2403)	
Exposure Classification	%(n/N)	
Mild	2.7 (67/2403)	
Moderate	74.8 (1798/2403)	
Severe	21.9 (527/2403)	
Follow up	%(n/N)	
Medical follow-up	33.1 (796/2403)	
Infectious diseases specialist assessment	30.3 (728/2403)	
Prophylactic medication	21.5 (517/2403)	

*IQR* interquartile range, *SD* standard deviation, *y* years

During follow up, it was found that 21.5 % received any type of prophylaxis for the HIV infection. However, prophylaxis in severe exposures was received in 94.6 % cases. The most used drug was zidovudine/lamivudine (AZT/3TC) in 87.1 %, followed by AZT/3TC plus lopinavir/ritonavir in 8.8 % and emtricitabine/tenofovir in 2 %. Other included medication regimens used were abacavir/lamivudine, atazanavir, efavirenz, and raltegravir. And 7 (0.3 %) patients received human immunoglobulin due to exposition to HBV.

Additionally, 30.3 % of the cases reported assessment by infectious diseases specialist, and 33.1 % had a medical follow-up 6 months after the event. However, in patients classified as severe exposure, 99.2 and 68.9 % were assessed by infectious diseases specialist and received medical follow-up, respectively. A case of seroconversion to HIV was presented during the study time period, representing 0.0004 % of all the followed cases. Furthermore, there was no other disease transmission diagnosed during the follow up time.

### Factors associated to the type of exposure

In bivariate analysis, mild exposure was significantly related to health sciences students (*p* = <0.00001). In contrast, being a nurse (*p* = 0.015), assessment by an infectious diseases specialist (*p* = 0.025), and presenting a medical follow-up during follow up (*p* = <0.00001) were associated inversely with mild exposure (Table 2).

**Table 2:** Bivariate analysis of event according to exposure classification

Mild exposure	Yes %	No %	OR (CI 95 %)	p value
Female	76.6	82.6	0.69 (0.35–1.37)	0.287
Urban area	80.6	69.5	1.82 (0.99–3.36)	0.051
Physician	6.0	14.1	0.39 (0.14–1.07)	0.071
Student	34.3	12.5	3.66 (2.18–6.15)	<0.00001
Nursing	32.8	47.9	0.53 (0.32–0.89)	0.015
Bacteriology	4.5	4.9	0.91 (0.28–2.92)	1.000
Odontology	7.5	7.3	1.03 (0.41–2.56)	0.814
Surgical Instrumentation	6.0	2.5	2.49 (0.88–7.08)	0.092
Cleaning personnel	9.0	9.6	0.93 (0.40–2.17)	0.861
Respiratory therapy	0.0	1.2	1.03 (1.02–1.04)	1.000
Infectious diseases specialist assessment	17.9	30.7	0.49 (0.26–0.93)	0.025
Prophylactic medication	0.0	22.1	1.04 (1.03–1.05)	<0.00001
Medical follow-up	11.9	33.9	0.26 (013–0.56)	<0.00001
Moderate exposure	Yes %	No %	OR (CI 95 %)	p value
Female	82.7	81.4	1.90 (0.83–1.43)	0.525
Urban area	70.1	69.0	1.05 (0.86–1.29)	0.627
Physician	15.5	8.9	1.88 (1.38–2.56)	<0.00001
Student	12.3	15.5	0.77 (0.59–0.99)	0.048
Nursing	48.6	44.1	1.20 (0.99–1.45)	0.056
Bacteriology	4.9	4.9	1.01 (0.66–1.55)	0.968
Odontology	7.0	8.2	0.83 (0.59–1.18)	0.298
Surgical Instrumentation	2.8	2.0	1.38 (0.73–2.61)	0.320
Cleaning personnel	7.5	16.0	0.42 (0.32–0.56)	<0.00001
Respiratory therapy	1.4	0.3	4.15 (0.98–17.58)	0.041
Infectious diseases specialist assessment	10.7	90.1	0.13 (0.01–0.02)	<0.00001
Prophylactic medication	0.0	87.0	24.48 (19.67–30.47)	<0.00001
Medical follow-up	23.7	62.5	0.19 (0.15–0.23)	<0.00001
Severe exposure	Yes %	No %	OR (CI 95 %)	p value
Female	82.0	82.5	0.96 (0.73–1.28)	0.068
Urban area	67.6	70.5	0.87 (0.71–1.08)	0.200
Physician	9.3	15.2	0.57 (0.42–0.79)	0.001
Student	13.1	13.1	0.99 (0.75–1.33)	0.987
Nursing	45.5	48.1	0.90 (0.75–1.10)	0.308
Bacteriology	4.9	4.9	1.01 (0.64–1.57)	0.980
Odontology	8.3	7.0	1.21 (0.85–1.73)	0.288
Surgical Instrumentation	1.5	2.9	0.52 (2.46–1.10)	0.082
Cleaning personnel	16.9	7.5	2.50 (1.88–3.32)	<0.00001
Respiratory therapy	0.4	1.3	0.28 (0.07–1.19)	0.097
Infectious diseases specialist assessment	99.2	10.9	1065.13 (394.06–2879.05)	<0.00001
Prophylactic medication	98.1	0.0	188.50 (101.59–349.76)	<0.00001
Medical follow-up	68.9	23.3	7.28 (5.89–9.02)	<0.00001

*CI* confidence interval, *OR* Odds ratio

Factors significantly related to moderate exposure events were physicians (*p* = <0.00001), and respiratory therapists (*p* = 0.041); however, the confidence interval of the group of respiratory therapy included the unit (CI95% 0.98–17.58). Meanwhile health sciences student (*p* = 0.048), and cleaning personnel (*p* = <0.00001), infectious diseases specialist assessment (*p* = <0.00001), and medical follow-up (*p* = <0.00001) were inversely related (Table 2).

Factors related severe exposure events were cleaning personnel (*p* = <0.00001), as well as infectious diseases specialist assessment (*p* = <0.00001), receiving post-exposure prophylaxis (*p* = <0.00001), and medical follow-up (*p* = <0.00001). Contrary, there was an inverse relationship between being a physician (*p* = 0.001) (Table 2).

As a result of a multivariate analysis it was found in mild exposure events, being a health sciences student was related to presenting a mild exposure event (AOR 11.91, 95 % CI 5.13–27.61, *p* = <0.00001). In contravention, having a medical follow-up was inversely related (AOR 0.19, 95 % CI 0.05–0.64, *p* = 0.008) (Table 3).

**Table 3:** Multiple logistic regression of event according to exposure classification

Mild classification logistic regression model				
Variable	β	AOR	CI 95 %	p value
Constant	−3.397	0.020	-	<0.0000001
Gender (female)	−0.234	0.792	0.381–1.647	0.532
Student	2.477	11.901	5.129–27.613	<0.0000001
Nursing	0.388	1.475	0.624–3.487	0.376
Infectious diseases specialist assessment	−0.065	0.937	0.411–2.134	0.877
Medical control	−1.684	0.186	0.054–0.642	0.008
Moderate classification logistic regression model				
Variable	β	AOR	CI 95 %	p value
Constant	3.845	46.750	-	<0.0000001
Gender (female)	0.347	1.414	0.924–2.164	0.110
Physician	0.641	1.898	1.174–3.068	0.009
Student	−2.532	0.080	0.042–0.149	<0.0000001
Respiratory therapy	0.251	1.286	0.184–8.984	0.800
Infectious diseases specialist assessment	−4.708	0.009	0.006–0.15	<0.0000001
Medical follow-up	−0.406	0.667	0.473–0.939	0.020
Severe classification logistic regression model				
Variable	β	AOR	CI 95 %	p value
Constant	−6.328	0.002	-	<0.0000001
Gender (female)	−0.362	0.697	0.416–1.166	0.169
Physician	−0.613	0.542	0.322–0.911	0.021
Cleaning personnel	0.173	1.189	0.662–2.135	0.563
Infectious diseases specialist assessment	7.057	1160.887	285.559–4719.372	<0.0000001
Medical follow-up	0.604	1.830	1.275–2.626	0.001

*AOR* Adjusted Odds Ratio, *CI* confidence interval

Moderate exposure, in multivariate analysis was associated with being a physician (AOR 1.90, 95 % CI 1.17–3.07, *p* = 0.009). While, health sciences students was inversely related to moderate exposure events (AOR 0.08, 95 % CI 0.04–0.15, *p* = <0.00001). Also, infectious diseases specialist assessment (AOR 0.01, 95 % CI 0.01–0.15, *p* = <0.00001), and medical follow-up (AOR 0.67, 95 % CI 0.47–0.94, *p* = 0.020), (Table 3).

Severe exposure in multivariate analysis showed relationship with infectious diseases specialist assessment (AOR 1160.89, 95 % CI 285.56–4719.37, *p* = <0.00001), and the medical follow-up (AOR 1.83, 95 % CI 1.28–2.63, *p* = 0.001). Although, physicians were inversely related to this classification (AOR 0.54, 95 % CI 0.32–0.91, *p* = 0.021) (Table 3).

## Discussion

One of the most related factors to biohazard events in HCW is occupation; previously, different studies have reported that exposure to blood borne pathogens is more frequent in personnel that must manipulate vascular accesses or blood samples such as nursing personnel (nurses and nursing assistants), physicians, and laboratory personnel [7–20].

According to these findings in our registry it was clear that the most frequently related occupations were: nursing, physician and health sciences student. Nursing with 47.5 % (1141 registries), although this value is not as high as reported in Spain (61.6–78 %) [17], Poland (68 %) [12], and Egypt (66.7 %) [11]. However, a similar percentage is found in other countries as Turkey (44 %) [7], and Georgia (39 %) [9]. This variation may be due to the current record includes professions not considered by these studies as students, cleaning personnel, laboratory personnel, and not only physicians and/or nurses.

To our knowledge there are few studies that have attempted to perform an analysis according to the severity classification proposed by the CDC, in our study statistically significant associations were found with each category; it was found and association to mild exposure event and being a student, that could be related to the population that presented more events with blunt needle (46.3 %) and mucosal splash (22.4 %) that is generally associated to mild exposure.

Physicians were associated to moderate severity exposure events, possibly explained by main exposure mechanisms as blunt needle puncture (32.1 %) and hollow needle puncture (30.2 %) in this occupational group.

Contrariwise being a physician was inversely associated to severe exposure events since physicians only represented 9.2 % of the cases. An interesting finding was that despite it was not evident an association in the logistic regression model, being part of the cleaning personnel was associated to severe exposure events, were 96.6 % of the reported events were associated to hollow needle puncture or unspecified needle puncture with unknown source.

A study including different occupational groups by Montufar-Andrade F, et al. [20]. conducted in Colombia, informed the occupational exposure in nursing (22.9 %), cleaning personnel (21.7 %), students in formation in 14.3 %, and physicians in 4.2 %, similarly to those found in our study. Additionally, In Turkey there was reported a 34 % of cleaning personnel in the exposure registries [7]. Regarding severe classification group, in Tunisia it was found that being part of the nursing personnel was a risk factor [14], and Colombian medical students in practice during surgical areas was associated to severe events [21].

Among factors reported previously associated to exposure to biological material in HCW are: working in the emergency room, working in the operating room, working in the ICU or in resuscitation area, inadequate illumination system, age under 25 years, lack of experience, workload >40 hours/week, lack of training in infection risk, and inconsistency in the use of gloves [16, 22–25]. Even, in some cases the use of gloves was only present in 47 % of the events [26]. Additionally, there was an association in HCW as students in formation, especially during surgical practices [21, 27–31], gynecology and obstetrics [21, 27–32], anesthesiology [31], internal medicine [27], or in the emergency room [29]. Those who were in surgical areas had the presence of severe exposure events [21], and nearly 20 % did not register the use of protective materials [28]. In the United Kingdom it was found that most physicians experienced events with suture (blunt) needles, while younger physicians presented exposure during laboratory sample recollection [33].

During the protocol period a case of HIV seroconversion occurred (0.0004 %); this case was recently described as the first case HIV seroconversion in a HCW from Colombia; the event occurred in 2005 and the patient did not receive post expositional prophylaxis [34], making clear the importance of a protocol for the proper and prompt attention of these events. On the other hand, no other additional infections were recorded in HCW.

Adherence is a major concern in HCW occupational exposure protocols with a large percentage of professionals that do not complete the prophylactic recommendations or fail to attend to follow-up and evaluation by an infectious diseases specialist [35, 36]; in an analysis of all the reports it was noticed that only 21.5 % received any type of post expositional prophylaxis with anti-retroviral medication, 33.1 % assisted to medical follow-up, and 30.3 % assisted to an appointment with infectious diseases specialist. However it is noteworthy that those patients classified as severe exposure 94.6 % received post expositional prophylaxis, 99.2 % assisted to medical control and 68.9 % were evaluated by an infectious diseases specialist.

Factors associated with poor adherence to protocol in HCW are described as poor risk awareness or lack of time, and these arguments could lead to an important sub-registry [4, 19]. In Colombia a study conducted by Tapias-Vargas LE et al. [29] in a group of residents, it was found that 31 % of this HCW did not report the event, and therefore did not received any guidance or comprehensive assessment.

There are several limitations of the current study consists in the retrospective character of the data and absence of sampling methodology. Similarly, other factors previously reported abroad could not be analyzed based on registries used. Although reports from several regions of the country where analyzed, including urban and rural areas, we cannot ensure representativeness of all the HCW population nationwide and underreport could be also considered since HCW must contact initially agencies in order to access to the program. Additionally, the transversal nature of the study does not allow making an inference of causality of the associated factors presented.

## Conclusion 

Occupational exposure events must be managed according to established protocols, but adherence failure was evident with the exception of severe exposure cases in this study and generally on published literature, so these results can promote the study of determinants in lack of adherence in Colombian HCWs. This study added evidence of relevance in the initial management of occupation, thus allowed a more specific assessment in HCWs with severe exposure classification.

## Abbreviations

AOR: adjusted odds ratio
CI: confidence intervals
HCW: healthcare workers
HBV: Hepatitis B Virus
HCV: Hepatitis C Virus
HIV: Human Immunodeficiency Virus
IQR: interquartile range
3TC: lamivudine
SD: standard deviation
AZT: zidovudine
